# Abiotic Stress Signaling and Responses in Plants

**DOI:** 10.3390/plants12193405

**Published:** 2023-09-27

**Authors:** Małgorzata Nykiel, Marta Gietler, Justyna Fidler, Beata Prabucka, Mateusz Labudda

**Affiliations:** Department of Biochemistry and Microbiology, Institute of Biology, Warsaw University of Life Sciences-SGGW, Nowoursynowska 159, 02-776 Warsaw, Poland; marta_gietler@sggw.edu.pl (M.G.); justyna_fidler@sggw.edu.pl (J.F.); beata_prabucka@sggw.edu.pl (B.P.); mateusz_labudda@sggw.edu.pl (M.L.)

## 1. Introduction

The responses of plants to stress factors are extremely elaborate. These responses occur at multiple levels, ranging from alterations in the molecular processes to structural changes in both the underground and aboveground parts of plants. The development of a comprehensive response to stress by plants is preceded by the activation of an effective system of signals. This Special Issue, under the title “Abiotic Stress Signaling and Responses in Plants”, is a miscellany of twelve papers (both original research and reviews) related to plant signaling under various abiotic stress factors.

## 2. Salt Stress

In the study of Wang et al. [1], the authors delved into the effects of salt stress on plant reproductive structures. After subjecting plants to high salt concentrations, there was a sharp decrease in water potential within these structures, leading to a notable increase in seed abortion. The researchers suspected that this seed failure might be associated with the accumulation of reactive oxygen species (ROS) in the plant’s ovules. Further investigation revealed genes responsible for ROS scavenging. Mutations in certain genes, such as iron-dependent superoxide dismutase (FSD2), ascorbate peroxidase (APX4), and three peroxidases (POD) (PER17, PER28, and PER29), resulted in a significant increase in seed failure under normal conditions. This study highlighted the complex relationship between ROS and seed development.

Similarly, in the study by Lu et al. [2], in which the effect of salinity on selected biochemical parameters in *Robinia pseudoacacia* seedlings was investigated, it was observed that NaCl treatment resulted in increased ROS accumulation. At a relatively low NaCl concentration (50 mM), an increase in the activity of antioxidant enzymes was observed, as well as an increase in the expression of genes related to the response to salinity, such as Na^+^/H^+^ exchanger 1 (*NHX1*) and salt overly sensitive 1 (*SOS1*). In turn, higher NaCl concentrations (100–200 mM) resulted in a decrease in antioxidant activity and the downregulation of the expression of the abovementioned genes, as well as a decrease in photosynthetic parameters and damage to the chloroplast structure. The authors indicated that *Robinia pseudoacacia* seedlings can tolerate low salt concentrations, while higher concentrations cause significant damage and metabolic disorders, resulting in a reduction in plant biomass.

This Special Issue also includes a paper on the Arabidopsis ABSCISIC ACID INTENSIVE 4 (ABI4) transcription factor (TF), which is involved in abscisic acid signaling and the related response to abiotic stresses. Maymon et al. (2022) [3] studied the influence of various factors on the stability of the ABI4 protein using transgenic Arabidopsis plants. It was observed that the level of ABI4 increased significantly in seedlings under NaCl stress. The obtained results indicate that ABAI4 is a highly unstable protein and is degraded by the 26S proteasome. In turn, the phosphorylation of serine 114 catalyzed by MAP kinases was responsible for the stabilization of the ABI4 protein. The authors suggested that the regulation of both gene expression and ABI4 protein levels is essential for the regulation of the activity of this key TF in abscisic acid signaling.

## 3. Water Stress

The study of Kim and Sung (2023) [4] focused on rice, a crop known for its high water consumption. To address this concern and enhance resource efficiency, the researchers examined the benefits of alternative wetting and drying (AWD) compared to continuous flooding in rice cultivation. AWD proved effective in acquiring soil nitrate, and it led to an abundance of amino acids in shoots, suggesting a rearrangement of amino acid pools to support protein production during the shift from vegetative to reproductive growth. This research suggested the potential advantages of AWD in rice production, emphasizing the need for the further exploration of form-dependent nitrogen metabolism and root development.

In the study of Zhang et al. (2023) [5], the researchers investigated the response of peach seedlings to drought stress and the role of lauric acid (LA). Their findings indicated that LA pretreatment mitigated the negative effects of drought stress on peach seedlings. LA played a role in preserving photosynthetic pigments, preventing the closing of pores, and increasing the photosynthetic rate. Additionally, LA reduced the levels of superoxide anion, hydrogen peroxide and malondialdehyde (MDA) by enhancing the activity of antioxidant enzymes like catalase (CAT), POD, superoxide dismutase (SOD), and ascorbate peroxidase (APX). RNA-Seq analysis revealed that LA affected the expression of genes associated with plant–pathogen interactions, phenylpropanoid biosynthesis, and calcium signaling pathways. These findings provided insights into the molecular mechanisms through which LA enhances drought resistance in peach trees.

The study of Xiong et al. (2022) [6] in maize explored the role of a class-II LBD TF called ZmLBD5 in response to drought stress. ZmLBD5 was found to regulate plant growth and drought tolerance. The overexpression of ZmLBD5 in Arabidopsis increased susceptibility to drought, which was characterized by higher rates of water loss and altered stomatal behavior. Importantly, ZmLBD5 also influenced the level of ROS, with increased activities of antioxidant enzymes like SOD and POD. This research highlighted the significance of ZmLBD5 in the plant’s response to drought stress, shedding light on its molecular mechanisms.

Waterlogging is an additional abiotic stress which inhibits aerobic respiration, resulting in the reduction of plant growth and significant yield losses in many plants. Waterlogging causes hypoxic conditions due to the slow diffusion of molecular oxygen in water, leading to various morphological and cellular acclimation responses. In the study of Li et al. (2023) [7] it was shown that waterlogging treatment reduced the levels of chlorophyll, soluble protein, soluble sugars, proline, and MDA in mulberry plants. Additionally, it decreased the activities of enzymes like APX, POD, and CAT. Waterlogging affected the rate of photosynthesis, stomatal conductance, and transpiration rate. It has been shown that the expression of 10,394 genes in total was changed under waterlogging, and photosynthesis-related genes such as cytochrome b6/f complex gene *petC* and photosynthetic electron transport gene *petE* were significantly downregulated. The research was conducted on mulberry cultivars with two propagation methods (cutting and grafting). Cutting groups were found to have a better ability to recover from waterlogging stress than grafted mulberries. These findings provide valuable insights into the underlying mechanisms of dual-method mulberry propagation in response to waterlogging stress and highlight the potential for developing waterlogging-tolerant mulberry cultivars.

## 4. Varia

Another important environmental stress is grazing, which significantly disturbs the growth and metabolism of plants, both through mechanical damage caused by foraging and trampling and the accumulation of feces. In the experiments conducted by Wang et al. (2022) [8], the influence of different grazing intensities on the transcriptome of *Taraxacum mongolicum* was examined. The greatest changes at the transcriptomic level of grazed plants compared to untreated plants concerned genes related to cell signaling and phytohormones, as well as those related to secondary metabolism and photosynthesis. Furthermore, it was observed that heavy grazing resulted in a more intense response at the transcriptomic level compared to light grazing. The authors indicated that the expression of genes related to secondary metabolism and photosynthesis may contribute to the increased stress tolerance of the studied species, and the obtained results provide important data on the molecular response to grazing.

Seed germination is a vigorous stage in the plant life cycle, and it begins with seed rehydration and imbibition. Due to the resumption of respiratory activity following imbibition, the oxygen content in the seed tissue is rapidly diminished. Therefore, the supply of the oxygen through the seed coat to the embryo becomes limited, which generates the hypoxic environment in the seed. The study of Jayawardhane et al. (2021) [9] on two species, hypoxia-tolerant rice and hypoxia-sensitive barley, showed differences in their metabolic activity, necessary for subsequent germination steps. It has been shown that the embryos of rice seeds have higher alcohol dehydrogenase activity, indicating more efficient anaerobic fermentation and an elevated nitric oxide (NO) level corresponding to a higher NO turnover rate via the phytoglobin–NO cycle. Both fermentation and NO turnover resulted in a higher ATP/ADP ratio in rice embryos prior to radicle protrusion, as compared to barley. Additionally, the activities of antioxidant enzymes (SOD, APX, monodehydroascorbate reductase, and dehydroascorbate reductase) in imbibed embryos were higher in rice than in barley, which corresponded to the reduced levels of ROS, MDA, and electrolyte leakage. In summary, the observation of metabolic changes in the embryos of two cereal species differing in tolerance to hypoxia can explain the adaptation of rice to low-oxygen environments.

This Special Issue also includes three review articles that analytically and critically summarize contemporary research in the area of plant signaling and response to abiotic stress. Each of them presents and analyzes research results from a different angle, the primary goal of which is to understand the induction processes and mechanisms that allow plants to survive unfavorable environmental and climatic conditions.

Thus, Jeyasri et al. [10] summarize the impact of important environmental factors causing abiotic stresses, such as atmosphere, soil, and water, on the growth and development of the world’s most important cereal species: rice, wheat, sorghum, and maize. In their article, they focus on the use of systems biology and advanced sequencing approaches in genomics to explain the mechanism of the response to abiotic stresses in cereals. The authors present a holistic approach, enabling an understanding the mechanism of response to abiotic stresses (bioinformatics and functional omics, gene mining and agronomic traits, genome-wide association studies, and TF family). In the article, they also highlight how progress in omics studies facilitates the identification of genes responsive to abiotic stresses and influences the understanding of the interactions between signaling pathways, molecular insights, novel traits, and their importance in cereal crops. The authors emphasize the need for further research to obtain information from integrated omics databases to understand the mechanisms of abiotic stresses, which gives hope for the development of plant production with a large spectrum of stress tolerance.

The review article by Nykiel et al. [11] focuses on the discussion of signal transduction pathways in cereals under the influence of factors such as drought, salinity, heavy metals, and attack by pathogens and pests, but also, which is especially worth emphasizing, the crosstalk between reactions in response to double stress. This work summarizes the latest discoveries regarding signal transduction pathways and integrates information available in the contemporary literature, which is an important starting point not only for the precise formulation of future research tasks that will lead to a full explanation of the mechanism of plant response to stress factors, but also for further progress in the creative breeding of stress-tolerant cultivars of cereals.

In turn, Radani et al. [12] focus in their article on the Helix–Loop–Helix (bHLH) plant family TFs, involved in responses to abiotic stress, representing one of the most important families of eukaryotic genes containing the highly conserved bHLH motif. These factors activate or repress the transcription of specific response genes and thus influence the response to abiotic stresses such as drought, climate change, mineral deficiencies, excessive salinity, and water stress, and the regulation of these factors is crucial in achieving a better control of their activity. With the use of figures, the authors present the structural features, classification, functions, and regulatory mechanisms of the expression of bHLH TFs at the transcriptional level by other pre- and post-translational components (ubiquitination, phosphorylation, and glycosylation) during their response to various abiotic stress conditions.

## 5. Summary

Taken together, the contributions presented in this Special Issue contribute significantly to our understanding of plant responses to environmental stressors, including signal transduction cascades, and indicates potential strategies to enhance the resilience, resource efficiency, and overall agricultural productivity of plants. As Guest Editors, we are thankful to all of the authors for choosing to publish their articles in our Special Issue. We also recognize and appreciate the engagement of the reviewers who reviewed all of the submitted manuscripts.

## Data Availability

Not applicable.
